# Elimination of the Actin-Binding Domain in Kelch-Like 1 Protein Induces T-Type Calcium Channel Modulation Only in the Presence of Action Potential Waveforms

**DOI:** 10.1155/2012/505346

**Published:** 2012-07-11

**Authors:** Kelly A. Aromolaran, Kelly A. Benzow, Leanne L. Cribbs, Michael D. Koob, Erika S. Piedras-Rentería

**Affiliations:** ^1^Neuroscience Graduate Program, Loyola University Chicago, Chicago, IL 60153, USA; ^2^Department of Neuroscience, Kennedy Center, Albert Einstein College of Medicine, Room 610, 1300 Morris Park Avenue, NY 10461, USA; ^3^Institute of Human Genetics, University of Minnesota, MMC 206, 420 Delaware Street SE, Minneapolis, MN 55455, USA; ^4^Office of Research Services, Room 4601, Building 102, 2160 S. First Avenue, Maywood, IL 60153, USA; ^5^Cellular and Molecular Physiology Department, Stritch School of Medicine, Loyola University Chicago, Room 4669, Building 102, 2160 S. First Avenue, Maywood, IL 60153, USA; ^6^Neuroscience Institute, Stritch School of Medicine, Loyola University Chicago, Room 4669, Building 102, 2160 S. First Avenue, Maywood, IL 60153, USA

## Abstract

The Kelch-like 1 protein (KLHL1) is a neuronal actin-binding protein that modulates calcium channel function. It increases the current density of Ca_v_3.2 (*α*_1H_) calcium channels via direct interaction with *α*_1H_ and actin-F, resulting in biophysical changes in Ca_v_3.2 currents and an increase in recycling endosomal activity with subsequent increased *α*_1H_ channel number at the plasma membrane. Interestingly, removal of the actin-binding Kelch motif (ΔKelch) prevents the increase in Ca_v_3.2 current density seen with wild-type KLHL1 when tested with normal square pulse protocols but does not preclude the effect when tested using action potential waveforms (AP). Here, we dissected the kinetic properties of the AP waveform that confer the mutant Kelch the ability to interact with Ca_v_3.2 and induce an increase in calcium influx. We modified the action potential waveform by altering the slopes of repolarization and/or recovery from hyperpolarization or by changing the duration of the depolarization plateau or the hyperpolarization phase and tested the modulation of Ca_v_3.2 by the mutant ΔKelch. Our results show that the recovery phase from hyperpolarization phase determines the conformational changes that allow the *α*_1H_ subunit to properly interact with mutant KLHL1 lacking its actin-binding Kelch domains, leading to increased Ca influx.

## 1. Introduction

Neuronal voltage-gated calcium channel function is central in processes such as neurotransmission and excitability, burst firing, intracellular signaling, and gene expression; thus, modulation of their activity can be physiologically relevant. The modulation of calcium channels by the actin cytoskeleton or actin-binding proteins (ABP) is not well understood. However, recently, we reported a novel mechanism of modulation of Ca_v_3.2 T-type channel *α*_1H_ subunit by the actin-binding protein Kelch-like 1 (KLHL1) [1].

KLHL1 is a neuronal ABP member of the Kelch superfamily that contains their two signature motifs: a BTB/POZ domain involved in protein-protein interactions and a Kelch *β*-propeller region involved in actin binding [2, 3]. This ABP is constitutively present in the nervous system, and it is involved in the modulation of neuronal structure and function; its genetic elimination results in loss of postsynaptic structures, a progressive loss of motor coordination and gait abnormalities in mice [4].

We have reported that KLHL1 is a modulator of voltage-gated calcium channels; it upregulates calcium current density of high- and low-voltage-gated channels [1, 5, 6]. We found that KLHL1 augmented the current density of Ca_v_3.2 (35% increase) but not Ca_v_3.1 [1]. Indeed, we demonstrated in biochemistry assays that KLHL1 co-precipitates with *α*_1H_  in membrane fractions obtained from the HEK 293 overexpression system and from mouse brains [1], corroborating that the ABP and *α*_1H_  directly interact. We found that this modulation is done by a novel mechanism that involves increasing the number of functional channels at the plasma membrane and by a discrete alteration of the kinetics of *τ*_deactivation_, resulting in increased calcium current density and calcium influx. The former effect occursvia enhanced channel reinsertion into the plasma membrane through the recycling endosome and requires the presence of polymerized actin [5].

**Figure 1:**
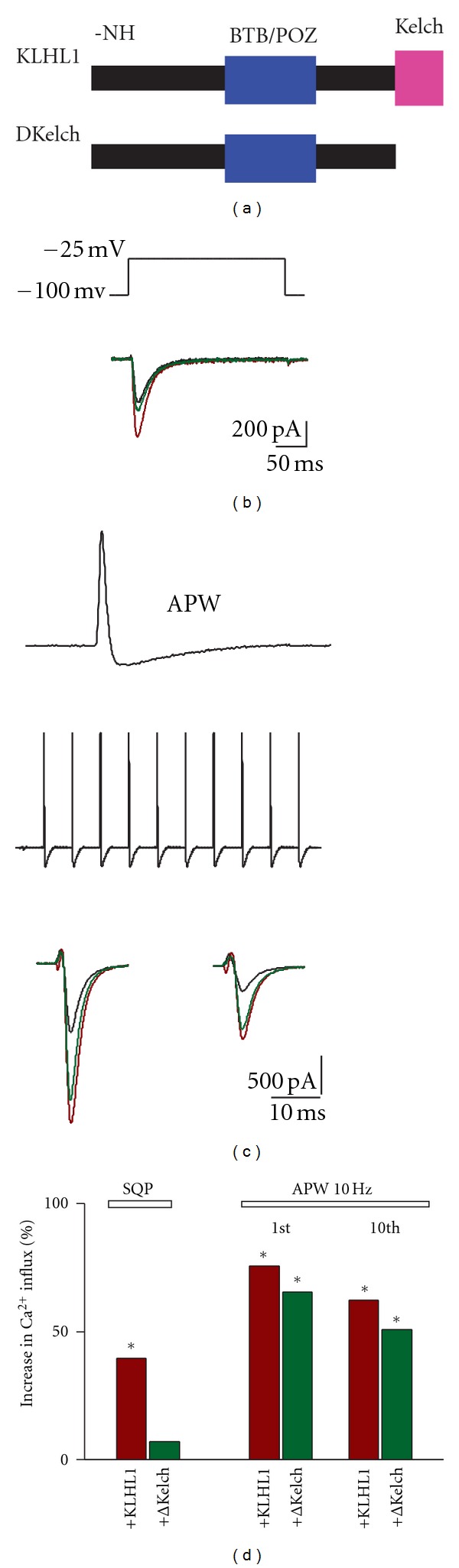
The wild-type KLHL1 actin-binding protein and the truncated mutant ΔKelch both modulate calcium influx through Ca_v_3.2 upon stimulation with action potentials. (a) Cartoon illustrating the overall structure of KLHL1 and ΔKelch; the amino terminus region (-NH), BTB/POZ, and Kelch domains are represented. (b) Example currents elicited by Ca_v_3.2 with a square pulse (SQP) from −100 to −25 mV under control conditions (black trace), or in the presence of KLHL1 (red trace) or ΔKelch (green trace). (c) Single-action potential (AP) or 10 Hz action potential waveform (APW) protocols used throughout this work. Examples of the current traces elicited for each condition are shown below, at the 1st and 10th spike during the APW train. (d) Percentage of increase in calcium influx with respect to control.

In our examination of the function of the motifs in KLHL1, we tested a truncated mutant lacking the Kelch domains (located at the carboxyl terminus of the protein) (ΔKelch construct, Figure 1(a)). Interestingly, the elimination of this actin-binding motif in KLHL1 resulted in the elimination of mostly all of the upregulation of Ca_v_3.2 by the ABP. However, when calcium influx was elicited with a single-action potential (AP) or an action potential train waveform protocol (APW), the ΔKelch mutant could still elicit the upregulatory effect [1]. Overall, these experiments indicated that the actin cytoskeleton is involved in the regulation of Ca_v_3.2 by KLHL1, as expected for an ABP, but they also suggested that additional regions in KLHL1 independent of the Kelch motif are involved in the direct interaction with the *α*_1H_ subunit. Here, we extend our studies to explore the specific properties of the action potential (AP) waveform that allow the interaction of the truncated KLHL1 mutant with Ca_v_3.2, resulting in increased calcium influx.

## 2. Material and Methods

### 2.1. Cell Culture and Transfection

Human embryonic kidney 293 cells stably transfected with the Ca_v_3.2 *α*_1H_ subunit were grown to 60% confluence and transfected using the calcium phosphate method [7] or polyethyleneimine (PEI) [8]. Cells were transfected with 1 *μ*g of human EGFP-KLHL1 cDNA or ΔKelch cDNA [1, 2]; EGFP cDNA was used to maintain equal transfection concentrations in control experiments.

### 2.2. Electrophysiology

Currents were recorded at 1–3 days after transfection using whole-cell patch clamp at room temperature as previously described [1, 5]. Currents were recorded using an Axopatch 200B amplifier (Axon instruments, Union City, CA), and data were acquired at 1 kHz and digitized at 10 kHz using the Digidata 1322 A analog-to-digital converter. Currents were recorded in an external solution containing (in mM) 5 CaCl_2_, 140 TEACl, 10 HEPES, and 10 glucose (pH 7.4, 300 mOsm). Pipettes pulled from borosilicate glass (Warner Instruments Inc., Hamden, CT) were filled with intracellular solution containing (in mM) 108 CsMeSO_3_, 4 MgCl_2_, 1 Cs-EGTA, 9 HEPES, 5 ATP-Mg, 1 GTP-Li, and 15 phosphocreatine-TRIS (pH 7.4, 280 mOsm). Cells with series resistance (*R*_*s*_) < 10 MΩ were used; *R*_*s*_ was compensated online (>80%). 

**Figure 2:**
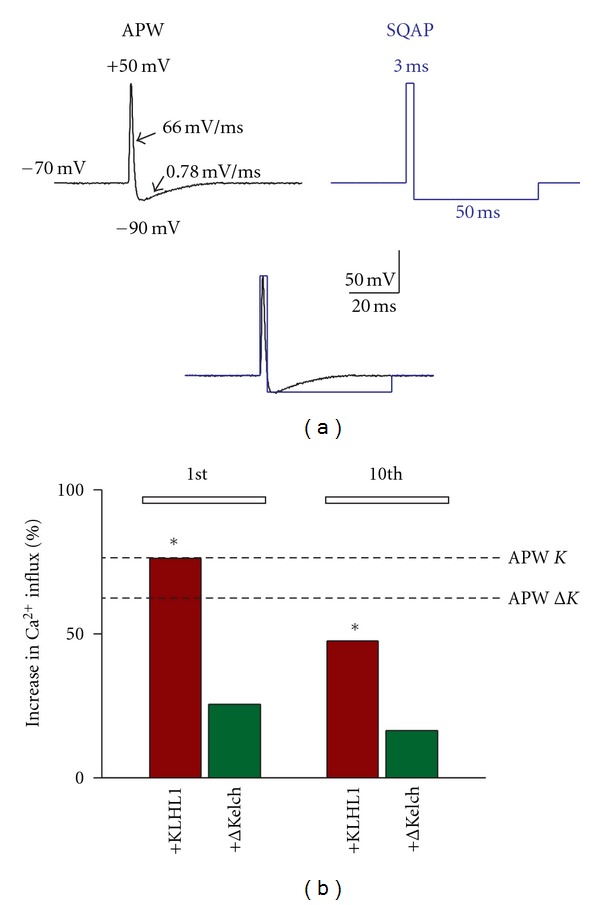
“Square” action potentials roughly similar to the original APW fail to enable upregulation of Ca_v_3.2 by ΔKelch. (a) Original APW waveform was used (left) and square action potential was tested (SQAP). The overlapped signals is shown for comparison. (b) Percentage of increase in calcium influx with the SQAP with respect to control; the magnitude of the changes induced by the original APWs are shown with dotted lines (*K* = KLHL1;  Δ*K* = ΔKelch); *P < 0.05.

Currents were elicited using a square pulse protocols from a holding potential (*V*_*h*_) = − 100 mV and depolarized for 150 ms to test potentials (*V*_*t*_) = −70 to +70 mV, in 5 mV increments. The action potential waveform (APW) consisted of a digitized action potential that had a resting potential of −70 mV, an upstroke of 116 mV/ms to a peak voltage of +50 mV, followed by a repolarizing downstroke at −65 mV/ms to a hyperpolarizing potential of −90 mV. The repolarization from afterhyperpolarization (AHP) slope was 0.78 mV/ms to resting conditions (Figure 2(a), left).

**Figure 3:**
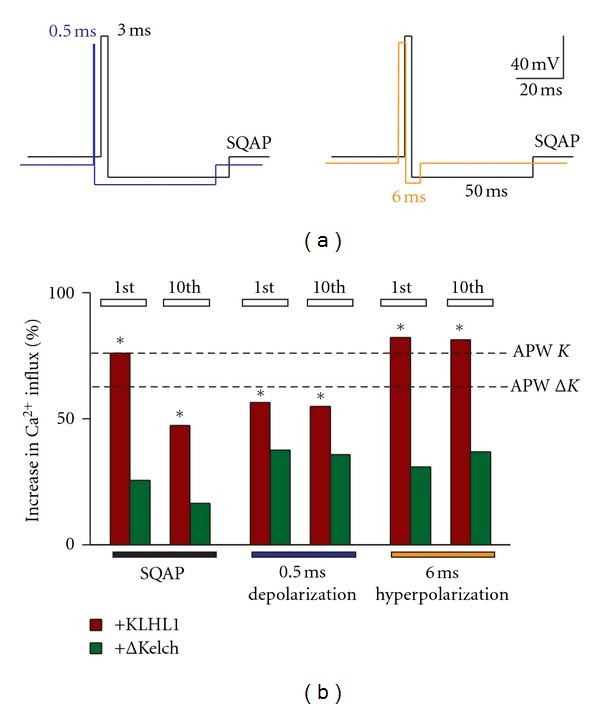
Changes in the duration of depolarization or hyperpolarization steps of the SQAP fail to enable upregulation of Ca_v_3.2 by ΔKelch. (a) Square AP with a shorter depolarization phase (0.5 ms, blue trace) or hyperpolarization phase (6 ms, orange trace). (b) Percentage of increase in calcium influx with the three protocols, with respect to control (action potential waveform, APW; the magnitude of the changes induced by the original APWs is shown with dotted lines (*K* = KLHL1;  Δ*K* = ΔKelch)); *P < 0.05.

**Figure 4:**
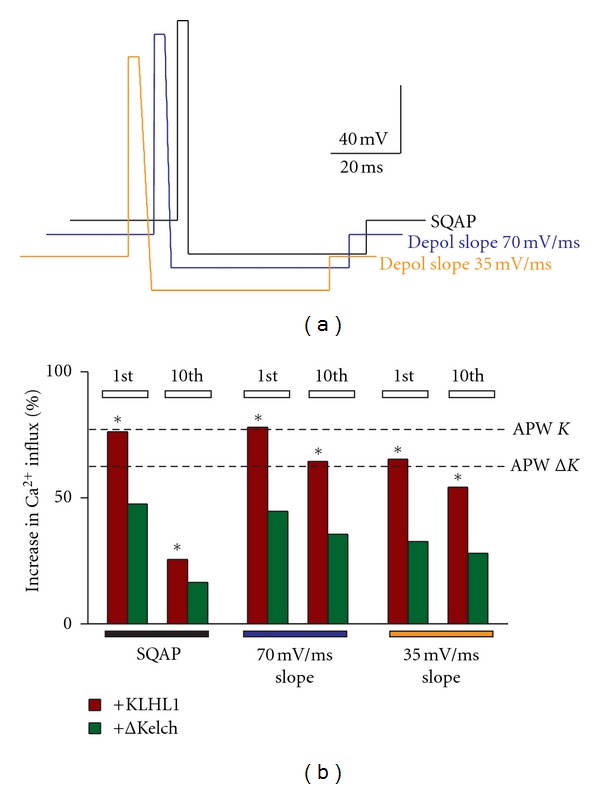
Changes in the repolarization slope of the SQAP fail to enable upregulation of Ca_v_3.2 by ΔKelch. (a) SQAP waveforms with instantaneous repolarization (black), midrange depolarization slope (70 mV/ms, blue), and slower repolarization (35 mV/ms, orange). (b) Percentage of increase in calcium influx with the three waveforms with respect to control; the extent of the changes induced by the original APWs is shown with dotted lines (*K* = KLHL1;  Δ*K* = ΔKelch); *P < 0.05.

**Figure 5:**
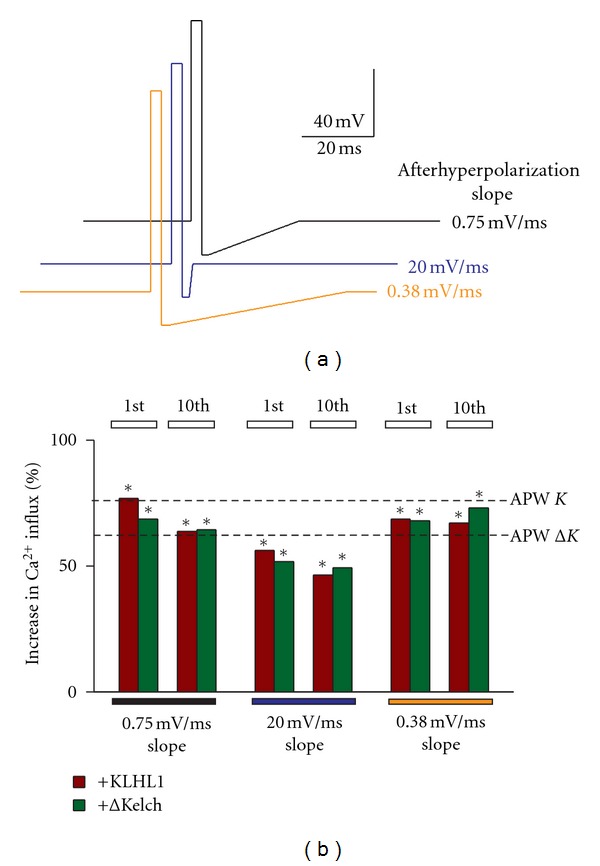
Changes in the afterhyperpolarization slope restore the ΔKelch effect, increasing Ca^2+^ influx through Ca_v_3.2. (a) SQAP with afterhyperpolarization slopes of 20 mV/ms (blue), 0.75 mV/ms (black), and 0.35 mV/ms (orange). (b) Percentage of increase in calcium influx with the three waveforms with respect to control; the magnitude of the changes induced by the original APWs is shown with dotted lines (*K* = KLHL1;  Δ*K* = ΔKelch); *P < 0.05.

**Figure 6:**
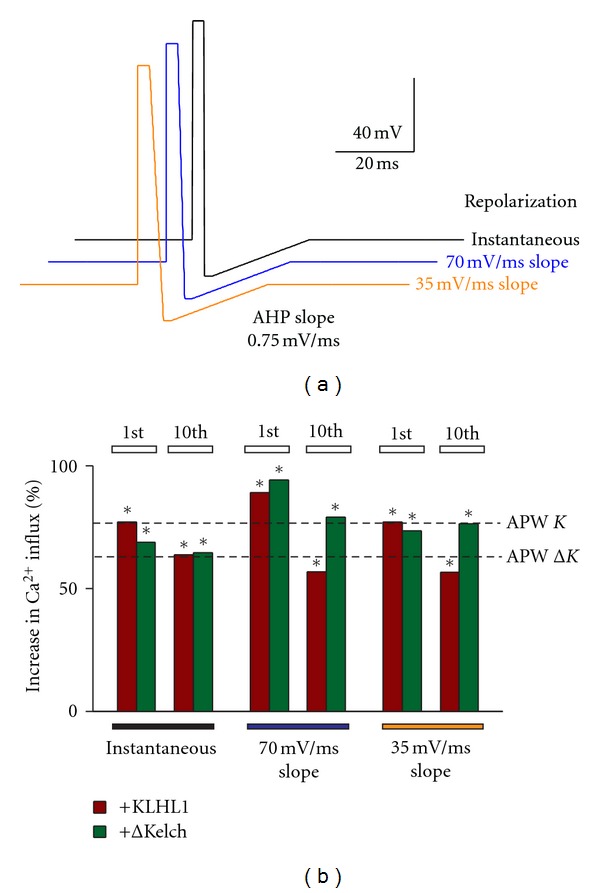
Changes in the repolarization slope in conjunction with an afterhyperpolarization slope synergize to enhance Ca^2+^ influx by ΔKelch. (a) Protocols with a sloping afterhyperpolarization ramp of 0.75 mV/ms were combined with repolarizing slopes of 70 or 35 mV/ms (blue and orange, resp.), and compared with a waveform with instantaneous repolarization (black). (b) Percentage of increase in calcium influx with the three waveforms with respect to control; the magnitude of the changes induced by the original APWs is shown with dotted lines (*K* = KLHL1;  Δ*K* = ΔKelch); *P < 0.05.

**Figure 7:**
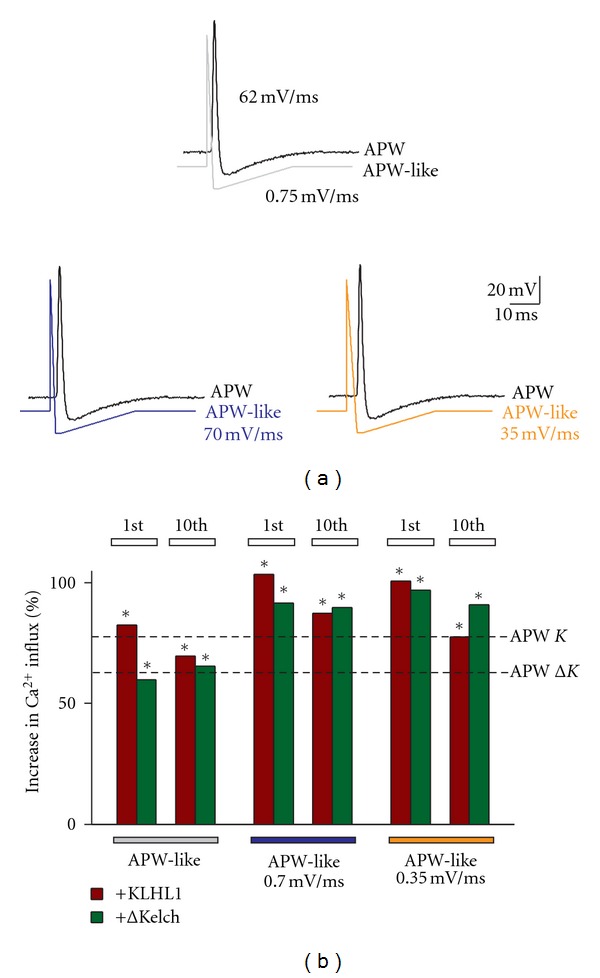
The APW-like stimulus produces an effect similar to an actual APW. (a) Stimulus (APW-like) created to replicate an actual APW (top) and APW-like stimuli with AHP slopes of 0.70 mV/ms and 0.35 mV/ms were compared. (b) Percentage of increase in calcium influx obtained with the stimuli shown above with respect to control; the magnitude of the changes induced by the original APWs is shown with dotted lines (*K* = KLHL1;  Δ*K* = ΔKelch); *P < 0.05.

The square pulse action potential waveform (SQAP) tested (Figure 2(a), right) consisted of a *V*_*h*_ = − 70 mV, instantaneous depolarization (120 mV/ms) to +50 mV for 3 ms, followed by instantaneous repolarization to −90 mV for 50 ms. Subsequent changes to the SQAP were as follows:

- changes in the duration of the depolarization step to +50 mV (0.5 ms, Figure 3(a), blue trace);
- changes in the duration of afterhyperpolarization to −90 mV (6 ms, Figure 3(a), orange trace);
- changes in the repolarization slope (from peak voltage, +50 mV) from instantaneous to 70 mV/ms (blue trace in Figure 4(a)) or to 35 mV/ms (orange trace in Figure 4(a));
- changes in the afterhyperpolarization slope (AHP slope) (from peak negative voltage = –90 mV) to resting potential at 20, 0.75 or 0.38 mV/ms slopes (Figure 5(a), blue, black and orange trace resp.);
- changes in both the repolarization and AHP slopes, resulting in the traces depicted in Figure 6(a), with the three repolarization slopes described in (c) and a 0.75 mV/ms afterhyperpolarization slope in all traces (Figure 6(a), black, blue and orange trace resp.);
- “action potential-like” waveforms, with repolarization ramp at 62 mV/ms and AHP slope ramp at 0.75 mV/ms (Figure 7(a), gray trace); repolarization ramp at 62 mV/ms and AHP slope ramp at 0.70 mV/ms (Figure 7(a), blue trace); repolarization ramp at 62 mV/ms and AHP slope ramp at 0.35 mV/ms (Figure 7(a), orange trace).

Total calcium influx was measured as the total charge elicited (in picocoulombs, pC) obtained by the integral of individual traces and normalized to cell size (pC/pF). Data were analyzed with Clampfit 9 software (Axon Instruments). Results are presented as mean ± SEM. Percentages of increase were calculated as the ratio of the experimental values (Ca_v_3.2 in the presence of ΔKelch or KLHL1) divided by the value of the Ca_v_3.2-mediated currents in the absence on KLHL1, expressed as percentage. Statistical significance was determined by *P* < 0.05 using Student's  *t*-test.

## 3. Results

### 3.1. Mutant KLHL1 Protein Lacking the Actin-Binding Domain (ΔKelch) Upregulates *α*_1H_  T-Type Currents Only with Action Potential Waveforms

The elimination of this actin-binding motif in KLHL1 results in the elimination of mostly all of the upregulation of Ca_v_3.2 by the ABP, as described in [1] and summarized in Figure 1(b), where currents elicited with a square pulse protocol (SQP) from −100 mV to −25 mV resulted in upregulated currents in the presence of KLHL1 (red trace) compared to controls (black trace), in contrast to the mutant ΔKelch (green trace). In contrast, when calcium influx was elicited with a single-action potential (AP) or an action potential train waveform protocol (APW), the ΔKelch mutant could still elicit the upregulatory effect (panel c). The data is summarized in panel (d), where it can be seen that the SQP protocol was overall less efficient at increasing calcium influx, and that an AP or APW protocol (in this case delivered at a frequency of 10 Hz) could still elicit calcium influx increase in the presence of the ΔKelch-truncated mutant. As expected, APWs delivered at 10 Hz or higher frequencies always elicited a concomitant current inactivation at the end of the train, as previously reported [1, 9]. These experiments suggest that additional regions in KLHL1, independent of the Kelch motif, are involved in the direct interaction with *α*_1H_; therefore in the following sections, we dissect the properties of the action potential (AP) waveform that are important for the interaction of the mutant ΔKelch with Ca_v_3.2.

### 3.2. “Square” Action Potentials Roughly Similar to the Action Potential Waveform (APW) Fail to Enable Upregulation of *α*_1H_  by ΔKelch

The upregulation of Ca_v_3.2 by KLHL1 or ΔKelch can be observed when the stimulus protocol is an AP waveform, irrespective of the frequency of delivery. We explored the consequences of modifying the AP on the effect of KLHL1 and ΔKelch on Ca_v_3.2-mediated calcium influx; APWs were delivered at 10 Hz throughout this study and are shown in all figures; however, only the values obtained for the first stimulus will be discussed for simplification purposes, as the effects are rather consistent at all frequencies throughout the study (up to 100 Hz, not discussed here). The main effect of changes in the stimulation frequency is the presence of current inactivation proportional to the frequency used [9, 10].

The original AP waveform was obtained from a hippocampal neuron and digitized for its use as the waveform protocol (see Methods). To dissect the AP components that allow calcium influx increase in the presence of ΔKelch, we first devised a rough “square action potential” or SQAP as depicted to the right in Figure 2(a), from the same  *V*_*h*_  as the AP waveform (APW) shown to the left, with an instantaneous upstroke to +50 mV followed by instantaneous repolarization back to −90 mV for 50 ms. Both stimuli are shown overlapped in Figure 2(a) (bottom) for comparison.

Delivery of an SQAP resulted in the expected increase in calcium influx by KLHL1 (76% increase (first stimulus), *n* = 19, *P* < 0.05), whereas ΔKelch only elicited a 25% increase, not statistically significant compared to controls (*n* = 38). The values reached with a conventional APW are denoted in the figure by the dotted lines for each KLHL1 and ΔKelch. Interestingly, ΔKelch not only failed to elicit a significant calcium influx increase with the SQAP stimulus, but the extent of the increase was much smaller than that obtained with a regular APW; this effect could also be observed in the 10th stimulus with KLHL1 (shown in Figure 1(d)). We then proceeded to modify the duration of depolarization of the waveform (at +50 mV) and the duration of the hyperpolarization at −90 mV, as seen in panel (a) from Figure 3.

### 3.3. SQAPs with Shorter Duration at Depolarized Potentials or Shorter Times of Hyperpolarization Fail to Enable Upregulation of Ca_v_3.2 by ΔKelch

The SQAP was modified by decreasing the time spent at +50 mV from 3 ms to 0.5 ms, as shown in Figure 3(a), left. The time spent at hyperpolarized potentials was also modified in a different protocol, which has reduced hyperpolarized potential duration (to −90 mV), from 50 ms to 6 ms (Figure 3(a), right).

As observed in panel (b), none of the manipulations rescued the effect of ΔKelch on Ca_v_3.2 as seen in figure (green bars). SQAP protocols elicited 25% of the influx of calcium by Ca_v_3.2 in the presence of ΔKelch (*n* = 38), and decreasing the depolarization time increased influx to 38% (*n* = 15), whereas decreasing the hyperpolarization time resulted in an increase of 31% (*n* = 22). In the case of KLHL1, all stimuli elicited significant increases in calcium influx compared to control (red bars). However, a decrease in the peak depolarization time resulted in a decrease of the effect of KLHL1 on *α*_1H_ as seen in Figure 3(b) (76% versus 57%, *n* = 19, 16, resp.). In contrast, a decrease in the hyperpolarization time to 6 ms resulted in calcium influx increase of 83% (*n* = 6). Incidentally, the latter stimulus was ideal to eliminate current inactivation after the 10th train (Figure 3(b), red bars), possibly providing more time for recovery from inactivation. Still, none of these changes enabled a calcium influx increase in the presence of ΔKelch thus, we next tested whether modification of the slope of repolarization has an effect on the calcium influx by ΔKelch.

### 3.4. SQAPs with Different Repolarization Rates Fail to Enable Upregulation of Ca_v_3.2 by ΔKelch

Two new stimuli with different repolarization rates were tested, an SQAP with a repolarization slope of 70 mV/ms in contrast to the instantaneous upstroke in the SQAP (120 mV/ms) (blue and black traces, resp., Figure 4(a)) and a waveform with a slower repolarization rate of 35 mV/ms. Overall, slowdown of the rate of repolarization did not rescue the effect of ΔKelch, as seen from the values represented in Figure 4(b). For the first stimulus, ΔKelch induced an increase in calcium influx of 25% (*n* = 38), as reported previously, whereas the altered waveforms elicited increases of 45 and 33%, respectively (*n* = 20, 19). The alteration of the slope rate resulted in calcium influxes of 78 and 65% above control for these stimuli in the presence of KLHL1 (*n* = 14, 14), compared to 76% with SQAP (*n* = 19); all these values are significantly higher than control. Incidentally, the calcium influx by Ca_v_3.2 after the 10th spike in the presence of KLHL1 appeared to be less inactivated when repolarization occurred with a slope as opposed to an instantaneous change in voltage (as in SQAP) (Figure 4(b)).

Given that neither the duration of depolarization, the duration of the hyperpolarization step, nor the slope of repolarization significantly altered the calcium influx by ΔKelch, we next targeted the AHP slope step in the action potential waveform.

### 3.5. The AHP Step in the APW Is a Major Determinant Allowing the Interaction between Ca_v_3.2 and ΔKelch and Thus Enabling Increased Calcium Influx by the Truncation Mutant

To determine whether the afterhyperpolarization slope step is important in the APW to enable the calcium influx increase seen with ΔKelch, we modified the SQAP protocol to include afterhyperpolarization ramps of varying slopes. These protocol waveforms are depicted in Figure 5(a), which included an afterhyperpolarizing slope of 0.75 mV/ms, a faster slope of 20 mV/ms, and slower slope of 0.38 mV/ms. As seen in the data shown in panel (b), changing the afterhyperpolarization step from a square pulse (seen in Figures 3 and 4) to a slope (Figure 5) enables the interaction between *α*_1H_-KLHL1. The protocol with an faster slope (20 mV/ms) enabled the least calcium influx, although its effect was still significant (57 and 52% for KLHL1 and ΔKelch) (*n* = 12, 12). On the other hand, the slower slope protocol (0.38 mV/ms) induced much higher calcium influx than the protocol with a slope of 0.75 mV/ms (69 and 68% increase for KLHL1 and ΔKelch, resp.), (*n* = 12, 14), comparable to the effect of the protocol with 0.75 mV/ms slope, clearly demonstrating that slower after-hyperpolarizing slopes also enable increased calcium influx through Ca_v_3.2 and more significantly allow the interaction between *α*_1H_ and ΔKelch.

We next combined the effects of the introduction of a repolarizing slope of 70 mV/ms, 35 mV/ms, or instantaneous repolarization, combined with an afterhyperpolarization slope of 0.75 mV/ms in all traces, as depicted in Figure 6(a). The latter AHP slope value was selected due to its similarity to the actual slope of the action potential (~0.78 mV/ms).

As seen in panel (b), all protocols elicited increased influx comparable with the control APW in the presence of KLHL1 (dotted lines, 76% increase); for the WT protein (KLHL1), influx was 77, 89, and 77% of control (*n* = 22, 18, 18). Similarly, ΔKelch elicited increases of 69, 95, and 74% in calcium influx (*n* = 24, 19, 20). Clearly, the afterhyperpolarization slope is paramount in enabling the interaction between the KLHL1 and *α*_1H_; introduction of a repolarizing slope induced the maximum increase in calcium influx observed thus far for ΔKelch.

Finally, we generated three protocols similar in shape to the original APW recorded from hippocampal neurons (Figure 7(a)). An APW-like protocol was generated with a repolarizing slope of 62 mV/ms (compared to 66 mV/ms of the original APW) and an afterhyperpolarization slope of 0.75 mV/ms (compared to 0.78 mV/ms). We produced another two waveforms with slight variations from the original: an APW-like protocol with a repolarizing slope from hyperpolarization of 0.70 mV/ms and a second waveform with a slower slope of 0.35 mV/ms. The results from these experiments are shown in Figure 7(b), which shows an enhancement of calcium influx concomitant with the slowdown of the afterhyperpolarization slope. For KLHL1, calcium influx was 83, 104, and 101% (*n* = 9, 19, 19), and similarly, ΔKelch enhanced Ca influx by 60, 92, and 97% (*n* = 12, 22, 22), corroborating the original APW data.

The experiments in this section clearly establish the importance of the AHP phase of the action potential for *α*_1H_  calcium influx. Moreover, this step allows the interaction between the mutant KLHL1 lacking the actin-binding Kelch motifs with the channel; this suggests that the deactivation step of *α*_1H_ channels is crucial to determine the interaction of the channel with the mutant ABP.

## 4. Discussion

The Kelch domain in KLHL1 is vital in the modulation of Ca_v_3.2 channels by KLHL1. In the absence of the *β*-propeller (ΔKelch), KLHL1 can still colocalize and interact with *α*_1H_ and elicit changes in Ca_v_3.2 calcium influx, albeit only with action potential stimuli [1]. Thus, KLHL1 requires its actin-binding Kelch domain to exert its effect on Ca_v_3.2. However, elimination of this domain uncovered another weaker interaction site between the channel and KLHL1. This interaction could only be observed during stimulation with action potentials. We investigated the properties within the action potential waveform that enable the interaction between *α*_1H_ and KLHL1.

Action potential waveform stimuli have been used to identify the specific contribution of calcium and other ionic currents during the physiological stimulus [11–13] and to determine how the currents' biophysical properties influence this contribution [14–16], see [17]. The shape and duration of the action potential are essential in determining the magnitude of neuronal calcium influx, specially given the importance of Ca_v_3.2 function in physiological processes such as adrenaline release from chromaffin cells, GABA release from thalamic reticular neurons, and in pain sensation [18–20]. Here, we modified the duration of the depolarization and the afterhyperpolarization steps, the repolarization slope, and the AHP slope. We found that a decrease of the plateau of depolarization tended to reduce Ca influx and that an increase in the length of hyperpolarization eliminated the frequency-dependent inactivation at 10 Hz, although neither of these changes rescued the effect of ΔKelch. Alteration of the slope of the repolarization phase did not rescue the effect of ΔKelch, although the slower rates of repolarization appeared to increase the calcium influx at the last spike of the stimulus [13]. 

In contrast, we established that the slope of AHP is the crucial step that enables the interaction between *α*_1H_ and ΔKelch. That this step resulted in an increase in calcium influx by Ca_v_3.2 is not a surprise, given the importance of tail currents in general in calcium entry [17], and specifically for Ca_v_3.2 with its slow kinetics of tail current deactivation. Thus, the transition step from open to closed state is necessary in the conformational change required to enable the interaction of regions in KLHL1—other than the Kelch domains—with the *α*_1H_ subunit. In summary, KLHL1 interacts with *α*_1H_ with at least two different regions of the protein; the main interaction involves the Kelch domains and requires stabilization by the actin cytoskeleton. This dominant interaction is responsible for the effects of KLHL1 on the channels, such as the increase in current density by increased endosomal recycling and channel number at the membrane, and the changes in kinetic in the time constant of deactivation. In the absence of this primary interaction, a secondary, more labile interaction is detected. This interaction is responsible for increases in calcium influx only seen when the protein is subjected to an AP stimulus; specifically, the afterhyperpolarization slope of the AP appears to be paramount in enabling this interaction, which suggests that the transition from open to closed state of the channel is necessary and allows a conformation change in *α*_1H_ conducive to its interaction with ΔKelch.
